# Dynamic follow‐up of smoldering multiple myeloma identifies a subset of patients at high risk of progression

**DOI:** 10.1002/ajh.26062

**Published:** 2020-12-19

**Authors:** Charlotte Gran, Vincent Luong, Johanna B. Bruchfeld, Johan Liwing, Gabriel Afram, Johan Lund, Saad Usmani, Evren Alici, Hareth Nahi

**Affiliations:** ^1^ Center for Hematology and Regenerative Medicine (HERM), Department of Medicine Karolinska Institutet Stockholm Sweden; ^2^ Department of Clinical Chemistry Karolinska University Laboratory Stockholm Sweden; ^3^ Haematology Center Karolinska University Hospital Stockholm Sweden; ^4^ Department of Hematologic Oncology and Blood Disorders Levine Cancer Institute/Atrium Health Charlotte North Carolina USA

To the Editor:

The risk of developing multiple myeloma (MM) from the smoldering multiple myeloma (SMM) is heterogeneous. To risk stratify SMM patients progressing to MM, several prediction models have been suggested. These prediction models have primarily been based on the level of monoclonal protein (MP) and the percentage of the bone marrow plasma cells (BMPCs) at diagnosis. The initial Mayo clinic prediction score defined MP ≥ 30 g/L and BMPC ≥ 10% as independent risk factors (RF) (Table S1 in Appendix S1), with a 5‐years risk of progression of 68% in patients with both RFs. In the updated 2014 IMWG diagnosis criteria, RFs (BMPCs ≥ 60%, FLCr ≥ 100 and >1 focal lesion on MRI) associated with ≥ 80% progression within 2‐years were classified as a myeloma defining event rather than ultra‐high risk SMM. Recent studies, including patients based on the 2014 diagnosis criteria, further proposed MP > 20 g/L, free light chain ratio (FLCr) > 20, and BMPCs > 20% as markers of progression. The cumulative increase of MP during follow‐up, a RF of evolving SMM, was initially described by Rosinol et al^1^ The risk progression associated with evolving MP has been observed in several retrospective studies,^2^, ^3^ but no prospective trials have confirmed this. In the prospective trial by Landgren et al it was observed that 12 (6%) of MGUS patients progressing to MM exhibited a phase of SMM prior to MM, and also identified an increase in iFLC as a risk factor for progression to MM.^4^ Recently, an SMM risk prediction model showed that the relative increase of involved FLC (eiFLC) was associated with a high risk of progression.^5^ However, FLC assessments are method dependent,^6^ with different reference ranges for FLC kappa and lambda and FLC ratio, rendering comparison between methods challenging. The cut‐offs for evolving biomarkers may thus differ depending on the assays used.

In this retrospective, exploratory study, we assessed biomarkers in 126 SMM patients (44 progressors, 82 non‐progressors) with a median follow‐up time of 4.5 years (Figure S1, Table S2 in Appendix S1). On average, MP and FLC were assessed every second month. Patients were stratified according to the previously published risk prediction models evaluating RFs at diagnosis (Table S1 in Appendix S1). We could validate MP > 20 g/L and BMPC > 20% at diagnosis as independent RFs for progression (Table 1). Based on ROC analysis, FLCr > 8 was identified as the optimal cut‐off, but was not an independent RF (Table 1). The median time to progression (TTP) was significantly shorter in patients with MP > 20 g/L and/or BMPCs > 20%, compared to low risk (Figure S2A in Appendix S1). This model cannot accurately discriminate between patients with one or two RFs during the first 2‐3 years. This might be due to the fact that RFs at diagnosis do not include the impact of evolving RFs. In total, 10 (8%) patients died without evidence of progression, with a median follow‐up time of 25 months (8‐110), and additionally, two (2%) patients were lost to follow‐up (at 12 and 33 months). In a competing risk analysis between the risk of death (without progress to MM) and risk of progression to MM, the cumulative incidence of progression at median follow‐up time was 40% compared to the incidence of death at 10%.

We analyzed our data using the previously reported risk prediction models, evaluating dynamic changes during follow‐up (Table S3 in Appendix S1). Several of the proposed evolving RFs were significant in univariate analysis. We further assessed the proposed evolving prediction model were by multivariate analysis. None of the evolving models could be validated in our cohort. One explanation could be the use of different assays in the in the assessment of biomarkers as well as the use of relative changes in the RFs. Therefore, in attempt to investigate the impact of absolute changes, the decline in Hb (within 12 months of SMM diagnosis) and an evolving FLCr (eFLCr), dFLC (edFLC), and/or MP (eMP), were evaluated in the current cohort. Note, ROC was performed to define the optimal cut‐offs (Table S4 in Appendix S1). Two dynamic RFs, assessed as time‐dependent variables, were identified as independent RFs in multivariate cox regression, eMP > 5 g/L and eFLCr > 4.5 (Table 1). Patients were stratified as low risk (no evolving RF, n = 57) or high risk (either one or both RFs, n = 57). High‐risk patients had a significantly shorter median TTP compared to low risk patients, *P* < .001 (Figure S2B in Appendix S1). An eFLCr and/or eMP was observed in 61 (48%) of the patients, 25 (30%) of the non‐progressors and 36 (82%) of the progressors. This increase was observed within the first 24 months of SMM diagnosis in 67% of patients, median time to increase 12.5 months for progress and 22 months for non‐progressors. Once an increase of eMP and/or eFLCr was observed the median time to MM progression was 5 months (2‐38 months). In a sub analysis by risk stratification at diagnosis, we observed that patients with an intermediate risk (MP > 20 g/L or BMPC > 20%) of progression at diagnosis and eMP > 5 g/L and/or eFLCr > 4.5 had significantly shorter TTP (median 42 months, 95% CI 9‐76) compared to patients with no evolving RF, where the median TPP was not reached, *P* = .001 (Figure S2C in Appendix S1).

**TABLE 1:** Univariate and multivariate cox proportional hazard analysis of prognostic risk factors at diagnosis and during follow‐up, of progression to MM

	No (%)	Univariate		Multivariate	
		HR (95%CI)	*P* value	HR (95%CI)	*P* value
At diagnose					
BMPCs					
≤20%	85 (68)	1	**.002**	1	**.007**
>20%	41 (33)	2.59 (1.41**‐**4.76)		2.48 (1.29**‐**4.79)	
MP					
≤20 g/L	55 (44)	1	**.003**	1	**.006**
>20 g/L	70 (56)	3.09 (1.48**‐**6.44)		3.45 (1.42**‐**8.35)	
FLCr					
≤8	72 (60)	1	**.033**	1	.223
>8	48 (40)	1.97 (1.06**‐**3.69)		2.51 (0.78**‐**2.92)	
Immunoparesis					
Absent	40 (34)	1	**.024**	1	.098
Present	79 (66)	2.55 (1.13**‐**5.76)		2.25 (0.86**‐**5.90)	
During follow‐up					
eMP					
≤5 g/L	71 (61)	1	**.001**	1	**.018**
>5 g/L	43 (39)	3.33 (1.68**‐**6.59)		2.40 (1.16**‐**4.97)	
edFLCr					
≤4.5	77 (68)	1	**<.001**	1	**.008**
>4.5	37 (32)	3.48 (1.79**‐**6.75)		2.57 (1.28**‐**5.19)	
eFLC					
≤20 mg/L	63 (55)	1	**.006**	1	.696
>20 mg/L	51 (45)	2.70 (1.34**‐**5.44)		1.21 (0.47**‐**3.14)	

*Note:* Immunoparesis was defined as the reduction, of at least one uninvolved immunoglobulin, below the lower normal limit (eg, IgG < 6.7 g/L, IgA < 0.88 g/L and IgM < 0.27 g/L). All significant values are in bold.

Abbreviations: BMPC, Bone marrow plasma cells; dFLCr involved, uninvolved free light chain ratio; edFLC, evolving involved‐uninvolved free light chain; eFLCr, evolving involved free light chain ratio; eMP, evolving monoclonal protein; MP, monoclonal protein.

In patients with MP ≤ 30 g/L (n = 92) at diagnosis, the presence of either one or both RFs were significantly associated with increased risk of progression (HR 3.36, *P* = .041 and HR 6.31, *P* < .001, respectively). Only 22 patients had MP > 30 g/L at diagnosis. These patients only had an increased risk of progression when both eMP > 5 g/L and eFLCr > 4.5 was observed during the follow‐up (*P* = .042). However, with the few number of patients in our cohort with MP > 30 g/L, these results need to be interpreted with caution and should be confirmed in a larger cohort.

Treatment of high‐risk SMM have been shown to prolong both the TTP and OS.^7^ Considering the risk associated with intervention, there is a need to accurately identify SMM patients at high risk. While several risk prediction models have been suggested, a low concordance has been observed between these studies.^8^ The utilization of different diagnostic criteria, IMWG 2003 or 2014, and different assays, renders the comparison of the risk prediction models challenging. Several studies have evaluated dynamic changes in FLC assessed by Freelite, however, to our knowledge; this is the first study assessing dynamic RF assessed by the N‐latex assay.

The retrospective nature of the present study has inherent limitations, such as the lack of bone marrow examinations at the follow‐up to evaluate both the increase in BMPCs and their phenotype. In addition, assessment of bone lesions was performed by low‐dose CT in all patients, but not MRI (only in 22%), which presumably could have led to the inclusion of treatment demanding MM in this cohort. Chromosomal aberrations were assessed by fluorescent in situ hybridization (FISH) in 79 (62%) of the patients (Table S5 in Appendix S1). We could not observe that previously reported cytogenetic RFs were associated with increased risk of progression, further interpretation of FISH results mandate caution due to the lack of a complete profile in all patients.

Several studies have shown that eMP is associated with a high risk of progression, however, with different cut‐offs of relative increase (Table S3 in Appendix S1). Furthermore, assessing a relative increase could include patients with a low absolute increase in patients with MP < 30 g/L, that is., in patients with an MP of 10 g/L as a diagnosis value, the absolute increase correlation to the suggested relative increases in previous studies, 10%, 25%, and 64% would give increases of 1, 2.5, and 6.4 g/L, respectively. Studies assessing absolute increase, showed that eMP > 5 g/L is an independent RF, which is in concordance with our data (Table S3B in Appendix S1). The impact of eiFLC > 169% as a RF^5^ could not be validated in our cohort. In addition, we could demonstrate that absolute increase exhibited a better discriminatory ability compared to a relative increase. It could be argued that relative increases could overestimate the impact of a dynamic biomarker, particularly in patients with low levels at diagnoses.

Anemia is one of the hallmark symptoms in symptomatic MM. Hence, a decrease in hemoglobin during the first year of diagnosis as a RF of progression in SMM has been suggested in previous studies (Table S3B in Appendix S1). We could not confirm that the decrease of hemoglobin by ≥ 5 g/L during the first year of follow‐up as a RF. Moreover, this cut‐off could include values within the intra‐individual variation of hemoglobin.

The findings in our retrospective study support that dynamic changes in MP can identify patients at high risk of progression. In conclusion, BMPCs > 20% and MP > 20 g/L at diagnosis, were independent RFs for the progression. Moreover, eMP > 5 g/L and eFLCr > 4.5 were significant predictors of progression during the follow‐up. With a median TTP of 5 months after an evolving pattern is observed, patients with evolving RF should be closely monitored. However, as these findings are exploratory, they should be validated in future prospective studies. The observed high risk of progression in patients with either or both evolving biomarkers may advocate a closer monitoring of these patients as well as possibly inclusion in future prospective early intervention trials.

## CONFLICT OF INTEREST

The authors declare no conflict of interest.

## AUTHOR CONTRIBUTIONS

C.G and H.N. conceived the study and oversaw overall direction and planning. C.G, J.B.B and V.L. collected the data. C.G., E.A., J.B.B., S.U. and H.N. wrote the manuscript with input from all authors. C.G., J.L. and H.N. analyzed the data. E.A. and H.N. supervised the project. All authors critically revised the manuscript and approved the final version.

## Supporting information


**Appendix** **S1**: Supporting information.

## Data Availability

The data that support the findings of this study are available upon reasonable request from the corresponding author. The data are not publicly available due to privacy or ethical restrictions.
