# Supplementary material for: Dynamic follow‐up of smoldering multiple myeloma identifies a subset of patients at high risk of progression
Source: Am J Hematol. 2020 Dec 19;96(3):E63–5. doi: 10.1002/ajh.26062 (PMC7898535; doi:10.1002/ajh.26062)
Supplement: Supplementary file 1 — Appendix S1: Supporting information. [file AJH-96-E63-s001.docx]

**Methods**

*Study population*

The study proposal was approved by the Swedish Ethical Review Authority and was performed in accordance with the Helsinki declaration. Informed consent was not collected after approval by the Swedish Ethical Review Authority since the study did not entail any new treatment or examinations.

For this study, we used the Karolinska University laboratory’s dysproteinemia database, including biomarker samplings from September 1, 2009, until April 1, 2017, including 4756 individuals, to identify patients with an SMM diagnosis, according to the revised 2014 IMWG diagnosis criteria for SMM(1), and assessment of MP and sFLC within 30 days of SMM diagnosis, supplemental figure 1. Assessment of MP and sFLC were required to be available within 7 days of each other. The electronic medical records for all patients included in the study were reviewed for demographics, imaging records at SMM diagnosis, date of MM progression, as well as to be able to exclude patients with other plasma cell disorders or hematological disorders. All included patients met the IMWG 2014 criteria of SMM: MP≥ 30g/L and/or clonal BMPC 10-60% and absence of end-organ damage and/or myeloma defining events(1). Patients without at least three serial measurement of MP and sFLC points prior to MM progression or last follow-up were excluded. In case of progression to MM, MP and sFLC values within 6 months before date of progression were censored, to avoid bias concerning a delay in MM diagnosis

Clinical and laboratory parameters were collected and compiled from all available sampling occasions during the study period for each patient. We briefly included the percentage of clonal BMPC, MP heavy and light chain subtypes and levels, total immunoglobulin levels, sFLC and iFLC concentrations, and sFLC kappa/lambda ratios, hemoglobin, creatinine, serum albumin, calcium, and, when available, beta-2-microglobulin. We calculated the estimated glomerular filtration rates (eGFR) according to the LMREV-formula(2).

Serum FLC assays were conducted with latex-enhanced immunonephelometric assay (Siemens Healthcare GmbH, Erlangen, Germany). Difference FLC (dFLC) was calculated as the difference between involved and uninvolved FLC, and FLC ratio (FLCr) was computed as the ratio of involved/uninvolved FLC. Serum protein electrophoresis and immunofixation were performed with agarose gels on the Hydrasys/Hydrasys 2 platform (Sebia, Lisses, France). Total serum immunoglobulin (IgG, IgA, and IgM) concentrations were analyzed using immunoturbidimetric assay (Roche Diagnostics GmbH, Mannheim, Germany). Immunoparesis was defined as the reduction, of at least one uninvolved immunoglobulin, below the lower normal limit (e.g., IgG <6.7g/L, IgA <0.88g/L and IgM <0.27 g/L). Pathology reports from bone marrow aspirates from each patient were reviewed for plasma cell burden at diagnosis. In the cases where there was a discrepancy between the percentage of plasma cells in the bone marrow smear morphology compared to immunohistochemistry, the higher of the two percentages was chosen.

*FLC assays used*

With regards to the assessment of FLC, two major assays have been utilized for diagnostic purposes. The initially developed Freelite assay utilizes polyclonal antibody mixes for the detection of FLC, whereas N-Latex FLC utilize monoclonal antibodies for more reproducible detection and tighter diagnostic window. Comparative studies between Freelite and N-Latex FLC have shown discrepancies both in the absolute sFLC values and iFLCr (3-6). A similar phenomenon has been observed for MP between different assays where a lack of concordance for MP evaluation can be seen(7-9). In addition, the biological variance (CVi) of both MP and FLC should be taken into account when evaluating the dynamics of these possible factors for risk prediction(10-12). In the 2014 IMWG criteria, assessment of sFLC was recommended, based on studied utilizing the Freelite assay, with an emphasis on the crucial role of the iFLCr as myeloma defining event. The lower threshold of 30 (3), as well as 70 (13), has been proposed to be equivalent to iFLCr>100 for a MM and SMM population with N-latex FLC assay compared to Freelite, respectively.

#### Outcome, risk stratification and evolving biomarker definition

Time to progression (TTP) was defined as the time from date of SMM diagnosis (by revised 2014 IMWG criteria) to date of treatment demanding MM or censored at the last follow-up date. Previous published risk factors for progression were evaluated in the cohort, table S1A-B. Potential evolving biomarkers, including serum MP, sFLC, and Hb, were extracted from all available data-points during follow-up until the date of symptomatic MM or last follow-up. Evolving biomarkers were defined as the absolute and relative difference between the first sample at SMM diagnosis and the sample with the highest level a minimum of 6 months before MM diagnosis or at last follow-up. A second sample was required to confirm the evolving pattern. For each potential prognostic variable, receiver operating characteristic (ROC) curves, with the area under the curve (AUC), were used to evaluate the prediction reliability, specificity, and sensitivity. The cut-offs for each variable were assessed by the Youden index.

#### Statistical analysis.

IBM SPSS Statistics performed data analysis for Windows, version 26 (IBM Corp., Armonk, N.Y., USA). Continuous patient parameters were expressed as medians and interquartile ranges, and the Kruskal-Wallis test was performed. For categorical variables, the Pearson chi-2 test was used. Univariate Cox regression was performed to estimate the effect of prognostic factors with hazard ratios (CI threshold: 95% and p-value threshold <0.05) reported. Multivariate analyses, including all variables significant (P < 0.05) in the univariate regressions, were assessed using Cox proportional hazard regression. The Kaplan-Meier method and Log-rank tests were performed for group comparisons of TTP.

**Supplemental tables**

**Table S1A-B**

Overview of risk models incorporating risk factors at diagnosis for smoldering multiple myeloma.

A. overview of cohorts

**Table S1A**

|  | **Current study** | **Kyle et al 2007²(14)** | **Dispenzieri et al 2008 ²(15)** | **Waxman et al 2015 ²(16)** | **Sørrig et al 2015 ²(17)** | **Lakshman et al 2018 ¹(18)** | **Hajek et al 2020 ², ³(19)** | | **Mateos et al 2020 ¹(20)** |
| --- | --- | --- | --- | --- | --- | --- | --- | --- | --- |
|  | **n=126** | **n=276** | **n=273** | **n=135** | **N=321** | **n=421** | **CMG n=287** | **Heidelberg n=240** | **n=1996** |
| progress to MM, n (%) | 44 (35) | 158 (57) | 158 (57) |  | 61 (19) | 165 (39) | 149 (52) | 93 (39) | 815 (41) |
| Median age SMM, years | 67 (33-93) | 64 (26-90) | 64 (26-90) | 62 (n.r.) | 70 (63-79 IQR) | 65 (30-92) | 62 (43-81) | 66 (46-84) | 64 (56-72) |
| Gender male, n(%) | 61 (48) | 171 (62) | 171 (62) | n.r. (55) | 172 (54%) | 246 (58) | 126 (44) | 130 (54) | 978 (49) |
| Inclusion period/ years | 2002-2019 | 1970-1995 | 1970-1995 |  | 2005-2014 | 2003-2015 | 2007-2013 | 2003-2012 | 2004-n.r |
| Median follow up time, years | 4.5 | 6.1 | 6.1 | 4 | 1.9 | 6.2 | 2.4 | 2.5 | 3.0 |

**Table 1B**

B. Comparison of hazard ratios for risk models

|  | **no** | **Uni/Mva** |  | **no** | **Uni/Mva** | **5Y risk** |  |  |  |
| --- | --- | --- | --- | --- | --- | --- | --- | --- | --- |
| **Kyle et al 2007** |  |  |  | **Kyle et al 2007²** | | |  |  |  |
| BMPC ≥10% | 26 (21) | 0.947/1.09 |  | n.r. | */* |  |  |  |  |
| MP≥30g/L | 26 (21) | 2.08*/2.12* |  | n.r. | */* |  |  |  |  |
| low risk |  |  |  | 27 |  | 15% |  |  |  |
| intermediate risk |  |  |  | 143 |  | 43% |  |  |  |
| high risk |  |  |  | 106 |  | 69% |  |  |  |
| **Dispenzieri et al 2008** |  |  |  | **Dispenzieri et al 2008 ²** | | |  |  |  |
| BMPC ≥10% | 26 (21) | 0.95/0.85 |  | 90 (90) | .- /3.1* |  |  |  |  |
| MP≥30g/L | 26 (21) | 2.08*/2.36* |  | n.r. | .- /1.9* |  |  |  |  |
| FLCr >8 or <0.125 | 48 (40) | 1.97*/2.39* |  | 164 (60) | .- /1.9* |  |  |  |  |
| low risk |  |  |  | 81 (30) |  | 25% |  |  |  |
| intermediate risk |  |  |  | 114 (42) |  | 51% |  |  |  |
| high risk |  |  |  | 78 (29) |  | 76% |  |  |  |
| **Waxman et al 2015** |  |  |  | **Waxman et al 2015 ²** | | |  |  |  |
| BMPC ≥40% | 15 (12) | 2.00/1.87 |  |  | 2.72* /- |  |  |  |  |
| iFLCr ≥50 | 3 (3) | 0.58/0.45 |  |  | 4.57* /- |  |  |  |  |
| albumin ≤3.5 | 68 (55) | 1.70/1.68 |  |  | 3.38* /- |  |  |  |  |
| low risk |  |  |  |  |  | 16% |  |  |  |
| intermediate risk |  |  |  |  |  | 44% |  |  |  |
| high risk |  |  |  |  |  | 81% |  |  |  |
| **Sørrig et al 2015** |  |  |  | **Sørrig et al 2015 ²** | | |  |  |  |
| Immunoparesis ⁴ | 77 (64) | 2.55*/2.57* |  | 214 (69) | 3.7*/ 2.7* |  |  |  |  |
| MP≥30g/L | 26 (21) | 2.08*/1.93* |  | n.r. | 3.0 */3.3* |  |  |  |  |
| low risk |  |  |  | 90 |  | 5% |  |  |  |
| intermediate risk |  |  |  | 165 |  | 18% |  |  |  |
| high risk |  |  |  | 42 |  | 38% |  |  |  |
| **Lakshman et al 2018** |  |  |  | **Lakshman et al 2018 ¹** | | |  |  |  |
| BMPC >20% | 41 (33) | 2.76*/ 2.85* |  | 152 (34) | 2.79* / 2.28* |  |  |  |  |
| MP >20g/L | 70 (56) | 3.09* / 3.59* |  | 195 (47) | 2.07* / 1.56* |  |  |  |  |
| iFLCr>20 | 23 (19) | 1.17 / 1.02 |  | 125 (30) | 2.23*/ 2.13* |  |  |  |  |
| low risk |  |  |  | 143 (34) |  | 9.7% |  |  |  |
| intermediate risk |  |  |  | 121 (29) |  | 26.3% |  |  |  |
| high risk |  |  |  | 153 (37) |  | 47.4% |  |  |  |
| **Hajek et al 2020** |  |  |  | **Hajek et al 2020 ², ³** | | | | |  |
|  |  |  |  | **CMG cohort n=287** | | | **Heidelberg cohort n=240** | | |
| Immunoparesis ⁴ | 77 (64) | 2.55 * /2.88* |  | 117 (52) | 2.16* / 1.18* |  | 169 (71) | 2.75* / 1.84 |  |
| iFLCr >30 | 14 (12) | 1.18/1.04 |  | 41 (27) | 2.51* / 2.35* |  | 33 (28) | 4.34* / 3.76* |  |
| MP≥23g/L | 63 (50) | 2.15*/ 1.96 |  | 106 (38) | 2.01*/ 1.55 |  | 64 (35) | 4.32 */3.64* |  |
| 0 RF |  |  |  | 48 |  | 18.5 | 26 |  | 5.3 |
| 1RF |  |  |  | 44 |  | 20.9 | 34 |  | 7.5 |
| 2RF |  |  |  | 32 |  | 41.8 | 41 |  | 44.8 |
| 3RF |  |  |  | 15 |  | 78.7 | 12 |  | 81.3 |
| **Mateos et al 2020** |  |  |  | **Mateos et al 2020** | | |  |  |  |
| BMPC >20% | 41 (33) | 2.76*/ 2.85* |  |  | / 2.29* |  |  |  |  |
| MP >20g/L | 70 (56) | 3.09* / 3.59* |  |  | / 2.07* |  |  |  |  |
| iFLCr>20 | 23 (19) | 1.17 / 1.02 |  |  | / 2.66* |  |  |  |  |
|  |  |  |  |  |  | 2Y risk |  |  |  |
| low risk |  |  |  | 522 (38) |  | 6% |  |  |  |
| intermediate risk |  |  |  | 445 (33) |  | 18% |  |  |  |
| high risk |  |  |  | 396 (29) |  | 44% |  |  |  |

**1;** IMWG 2014 SMM diagnosis criteria, **2;** IMWG 2003 SMM diagnosis criteria, **3;** Hajek et al 2020 two cohorts analysed as training and validation cohort. Results are presented per cohort, **4**; Defined as one of both polyclonal Ig decreased below the lower reference level. MP denotes monoclonal protein, iFLC involved free light chain, BMPCs bone marrow plasma cells, sFLC serum free light chain

**Table S2**

| Table S2 Patient characteristics | | | | |
| --- | --- | --- | --- | --- |
|  | **All** | **SMM non progressors** | **SMM progressors** | **P value** |
|  | **n=126** | **n=82** | **n=44** |  |
|  |  | ***no. patients (%)*** | |  |
| Gender, male no. (%) | 61 (48) | 42 (51) | 19 (43) |  |
| Age, years median (range) | 70 (33-93) | 70 (33-93) | 70 (37-88) | 0.34 |
|  |  | ***Median (IQR)*** | |  |
| Albumin | 35 (32-38) | 35 (33-38) | 35 (32-37) | 0.17 |
| B2m | 2.4 (1.9-3.2) | 2.4 (1.9-3.3) | 2.5 (1.9-3.1) | 0.70 |
| Hemoglobin, g/L | 126 (116-137) | 126 (117-137) | 126 (115-135) | 0.47 |
| Creatinine, μmol/L | 76 (65-97) | 78 (65-98) | 76 (63-87) | 0.40 |
| eGFR, mL/min/1,73 m² | 70 (56-81) | 69 (55-79) | 72 (61-85) | 0.21 |
| Calcium, mmol/L | 2.32 (2.25-2.41) | 2.32 (2.26-2.42) | 2.32 (2.25-2.39) | 0.67 |
| MP, g/dL | 23 (16-30) | 20 (12-26) | 29 (21-32) | **<0.001** |
| dFLC, mg/L | 61 (12-152) | 50 (9-152) | 88 (16-174) | 0.24 |
| BMPCs %, median (range) | 18 (1.5-60) | 17 (1.5-60) | 20 (2.5-29.5) | 0.39 |
|  |  | ***no. patients (%)*** | |  |
| Heavy chain type |  |  |  | 0.75 |
| IgG | 101 (80) | 65 (79) | 36 (82) |  |
| IgA | 24 (19) | 16 (20) | 8 (18) |  |
| Other | 1 (1) | 1 (1) | 0 (0) |  |
| Light chain type |  |  |  | 0.09 |
| lambda | 44 (35) | 33 (40) | 11 (25) |  |
| kappa | 82 (65) | 49 (60) | 33 (75) |  |
| sFLC ratio |  |  |  | 0.53 |
| normal | 15 (21) | 18 (23) | 7 (18) |  |
| abnormal | 95 (79) | 62 (77) | 33 (83) |  |

Patient characteristics at SMM diagnosis. Patients with SMM progressing to MM compare to non-progressors differs at MP levels at diagnose. Median follow-up for the cohort was 4.5 years from SMM diagnoses. 44 (35%) progressed to treatment demanding MM in a median time of 21 months (range: 11-41). B2M denoted beta-2-microglibulin, eGFR estimated glomerular filtration rate, MP monoclonal protein, dFLC difference involved-uninvolved free light chain, BMPCs bone marrow plasma cells, sFLC serum free light chain

|  | **Current study ¹** | **Ravi et al 2016 ¹(21)** | **de Larrea et al 2018 ²(22)** | **Wu et al 2018 ²(23)** | **Atrash et al 2018 ¹(24)** |
| --- | --- | --- | --- | --- | --- |
|  | **n=126** | **n=190** | **n=206** | **n=273** | **n=134** |
| Inclusion period years | 2002-2019 | 1973-2014 | 1973-2012 | 2010-2015 | 2012-2017 |
| Median follow up time, years | 4.5 | 10.4 | 6.8 | 5.6 | 2.4 |
| Age, median (range) | 67 (33-93) | 64 (30-83) | 64 (32-92) | 60 (20-90) |  |
| Gender male, no (%) | 61 (48) | 104 (55) | 75 (36) | 143 (52) | 60 (45) |
| Progress to MM, no (%) | 44 (35) | 134 (71) | 107 (52) | 123 (45) | 23 (17) |
| Myeloma-defining event ¹ |  | | | | |
| negative MRI/CT, no (%) | 28 (22) / 126 (100) | (100)/- | n.r./n.r. | (24) / (24) | (100)/- |
| PBMCs >60%, no (%) | 0 (0) | 0 (0) | 8 (4) | 22 (8) | 0 (0) |
| iFLCr >100, no (%) | 0 (0) | 0 (0) | n.r. | 27 (15) | n.r. |

**Table S3A-B**

Overview and comparison of dynamic risk models for smoldering multiple myeloma.

S3A. overview of cohorts

S3B. Comparison of hazard ratios for risk models

|  | **Current study ¹** | |  |  |  |
| --- | --- | --- | --- | --- | --- |
| **Current study ³** | **no (%)** | **Uni/MVA** |  | **no (%)** | **Uni/MVA** |
| eMP >5g/L | 43 (38) | 3.33* / 2.40* |  |  |  |
| eFLCr >4.5 | 37 (32) | 3.48* / 2.57* |  |  |  |
| **Ravi et al 2016 ⁴** |  |  |  | **Ravi et al 2016 ¹** | |
| BMPC≥20% | 41 (33) | 2.59*/ 2.97* |  | 91 (48) | 4.41* / 3.37* |
| eHb | 65 (52) | 1.35 / 1.05 |  | 48 (25) | 8.25* / 5.86* |
| eMP | 36 (32) | 3.75*/3.71* |  | 58 (31) | 9.55* / 8.20* |
| **de Larrea et al 2018 ⁵** |  |  |  | **de Larrea et al 2018 ²** | |
| BMPC ≥20% | 41 (33) | 2.59*/ 2.20* |  | n.r. | 3.70*/ n.r. |
| immunoparesis | 77 (64) | 2.55* / 2.61* |  | 87 (53) | 2.20*/ n.r. |
| eMP | 75 (66) | 4.45* / 3.75* |  | 47 (25) | n.g. / 5.1* |
| **Wu et al 2018 ⁶** |  |  |  | **Wu et al 2018 ²** | |
| immunoparesis | 77 (64) | 2.55* / 2.91* |  | n.g. | 2.90* / 3.90* |
| eHb | 14 (11) | 1.10 / 1.21 |  | 35 (13) | 4.54* / 8.05* |
| eMP | 26 (23) | 1.89 / 1.26 |  | 33 (12) | 3.64* / 3.98* |
| eiFLC | 44 (39) | 2.60 */ 1.95 |  | 23 (9) | 3.02* / 2.84* |
| **Atrash et al 2018 ⁴** |  |  |  | **Atrash et al 2018 ¹** | |
| BMPC≥20% | 41 (33) | 2.59*/ 2.55* |  | n.r. | 2.27 ⁷ /2.74* |
| iFLCr ≥8 | 48 (60) | 1.97*/1.78* |  | n.r. | 2.79* /2.66* |
| eMP | 36 (32) | 3.75*/3.81* |  | n.r. | 3.90*/ 4.47* |

**1**; IMWG 2014 SMM diagnosis criteria **2**; IMWG 2003 SMM diagnosis criteria, FLCr denotes involved/uninvolved free light chain ratio, BMPCs bone marrow plasma cells, MP monoclonal protein. **3**; eMP defined as ≥5g/L increase from SMM diagnose up to 6 month prior to MM diagnose, eiFLCr defined as ≥4.5 increase in involved/uninvolved FLC ratios from SMM diagnose up to 6 months prior to MM diagnosis. **4**; eMP defined as ≥10% increase within 6 months of SMM diagnosis (if M protein ≥30g/L) or ≥25% increase within 12 months with a minimum increase of 5g/L and/or 500mg/dL, eHB defined as decrease of ≥5g/L withing 12 months of SMM diagnosis. **5**; eMP defined as ≥10% increase within 12 months of SMM diagnosis (if M protein ≥30g/L) or ≥10% increase within 3 years with progressive increase in each annual sampling (if M protein <30g/L. **6**; eMP defined as >64% increase of M-protein, eiFLC defined as >169% increase of iFLC (involved-uninvolved FLC) and eHb as >1.57g/dl decrease of Hb, all three evaluated within 12 months of SMM diagnose. **7** p=0.051. ***** significant HR

Table S4

Overview of ROC curve analysis for risk factors at diagnosis and during follow-up.

| Table supplemental 4. ROC curve analysis | | |
| --- | --- | --- |
| **Parameters at diagnose** | **AUC (95%CI)** | **P-value** |
| BMPC% | 0.55 (0.43-0.66) | 0.39 |
| MP | 0.74 (0.65-0.83) | <0.001 |
| dFLC | 0.60 (0.48-0.71) | 0.11 |
| FLCr | 0.63 (0.51-0.74) | 0.036 |
| HB | 0.54 (0.44-0.65) | 0.42 |
| Albumin | 0.57 (0.47-0.68) | 0.17 |
| B2M | 0.51 (0.40-0.64) | 0.77 |
|  |  |  |
| **During follow-up** |  |  |
| absolute change |  |  |
| eMP | 0.80 (0.72-0.89) | <0.001 |
| edFLC | 0.72 (0.62-0.82) | <0.001 |
| eFLCr | 0.76 (0.66-0.86) | <0.001 |
| eHB | 0.55 (0.44-0.65) | 0.40 |
| relative change |  |  |
| eMP | 0.71 (0.62-0.81) | <0.001 |
| edFLC | 0.69 (0.58-0.80) | 0.001 |
| eFLCr | 0.76 (0.66-0.87) | <0.001 |
| eHB | 0.55 (0.44-0.65) | 0.41 |

BMPCs denotes bone marrow plasma cells, MP monoclonal protein, dFLC involved-uninvolved free light chain, FLCr involved/uninvolved free light chain ratio, eMP evolving monoclonal protein, eFLCr evolving involved/uninvolved free light chain ratio, edFLC evolving involved-uninvolved free light chain, eHB evolving haemoglobin

Table S5

Fluorescent in situ hybridisation at diagnoses. Hazard ratio assessed as univariate cox regression.

| FISH | HR (95%CI) | P value | Median (95%CI) | P-value |
| --- | --- | --- | --- | --- |
| Hyperdipoidy |  | 0.31 |  | 0.31 |
| no (n=40) | 1 |  | 46 (0-106) |  |
| yes (n=39) | 1.41 (0.73-2.27) |  | 38 (14-62) |  |
|  |  |  |  |  |
| t(4;14), del17p and/or hyperdiploidy | | 0.22 |  | 0.21 |
| no (n=27) | 1 |  | 86 (21-151) |  |
| yes (n=52) | 1.60 (0.76-3.37) |  | 38 (18-58) |  |

**Figure S1**

Consort diagram of cohort
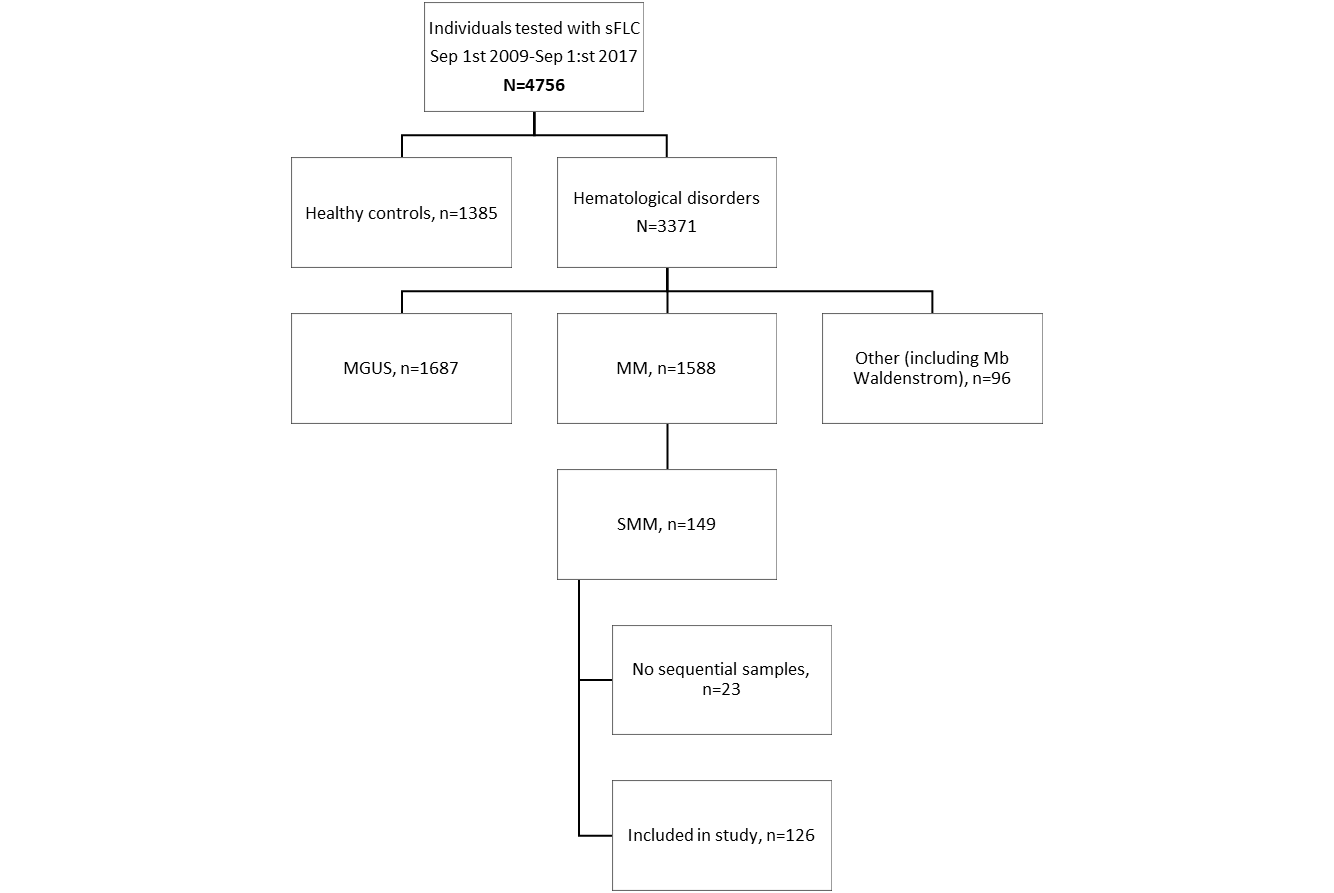


**Figure S2**

Risk stratification of smoldering multiple myeloma according to risk factors at diagnose or during the follow-up. Kaplan-Meier curves showing time to progression (TTP).

**Figure S2A**

Stratified by risk factors (RF) at diagnosis, bone marrow plasma cells percentage (BMPCs) >20% and/or monoclonal protein (MP)>20g/L, as low risk (none), intermediate risk (one risk factor), or high-risk (both).


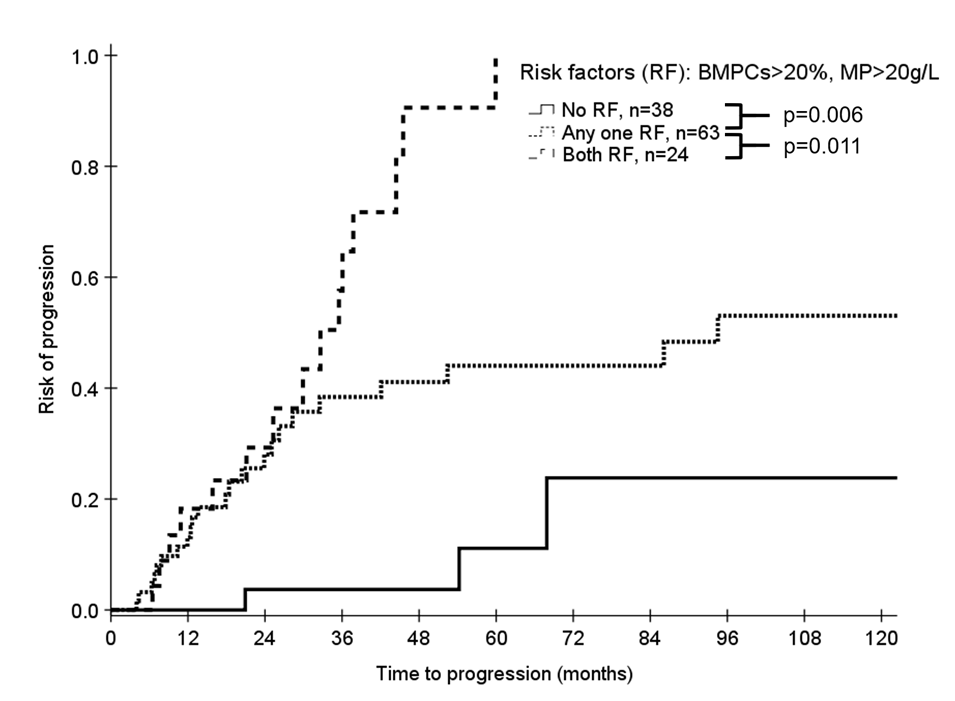


**Figure S2B**

Stratified by evolving risk during the follow-up, evolving monoclonal protein (eMP) >5g/L and evolving involved/uninvolved free light chain ratio (eFLCr) >4.5. Patients with both risk factors had a median TTP of 32 months (95% CI 20-45 months), with 68% progressed within two years of follow-up


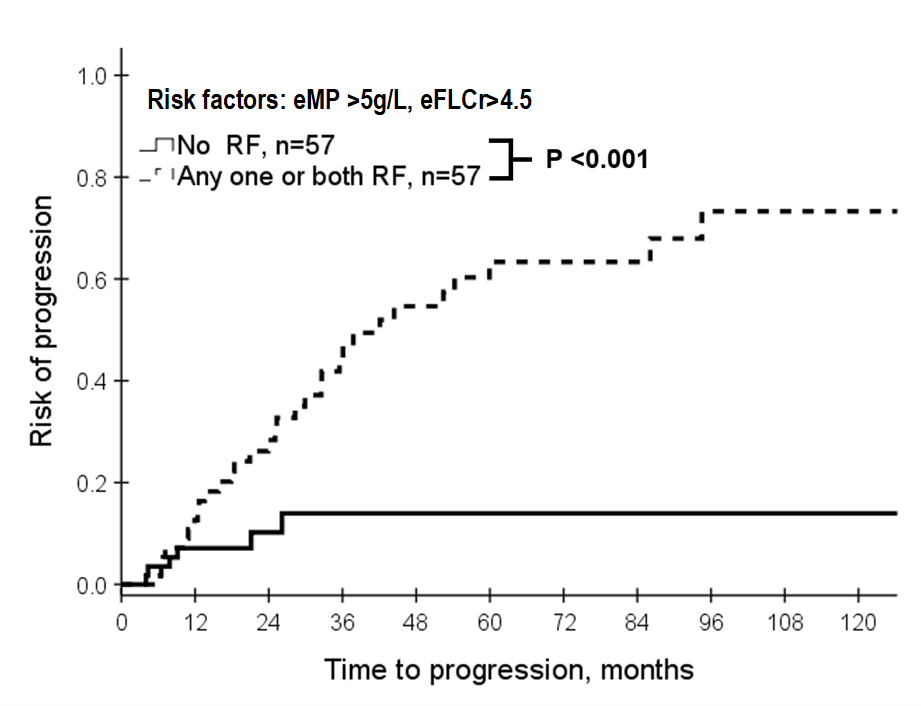


**Figure S2C**


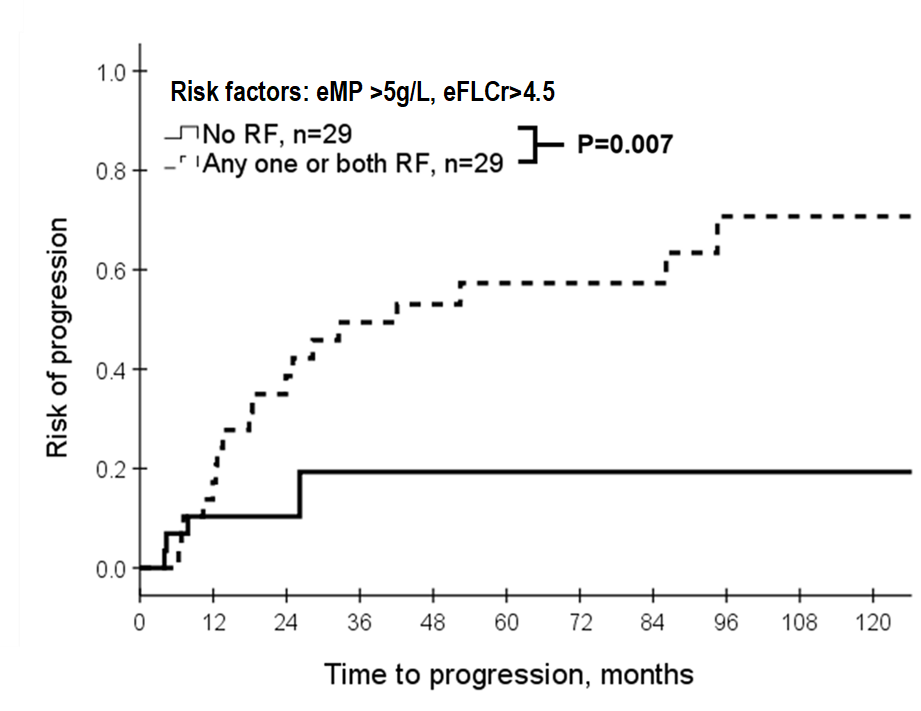

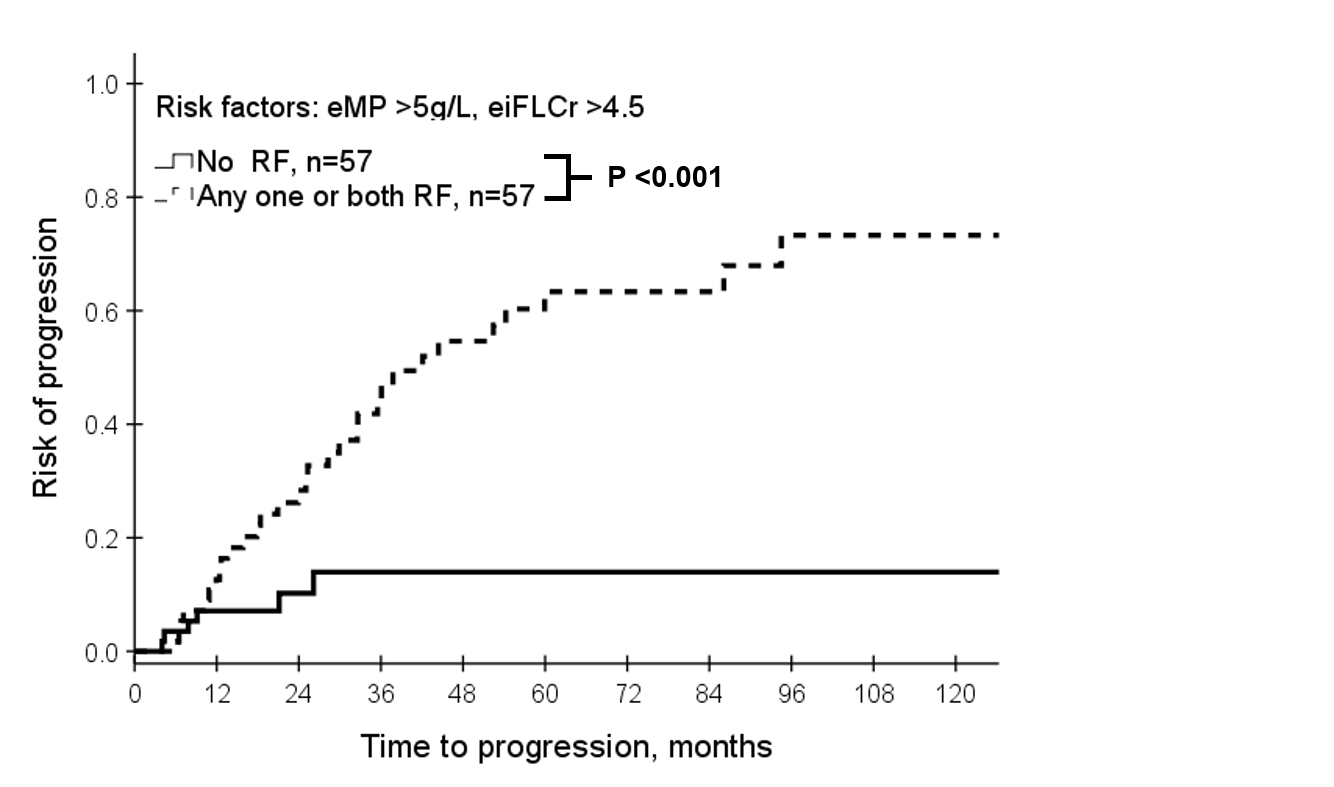
Stratification of patients with intermediate risk, either BMPCs >20% or MP>20g/L at diagnosis, and eMP >5g/L and/or eFLCr >4.5 during the follow-up. Patients with eMP >5g/L and eiFLCr >4.5 had significantly shorter TTP (median 28, range 6-51 months) compared to patients with no or one evolving risk factor, median TPP was not reached, p=0.001

References

1. Rajkumar SV, Dimopoulos MA, Palumbo A, Blade J, Merlini G, Mateos MV, et al. International Myeloma Working Group updated criteria for the diagnosis of multiple myeloma. Lancet Oncol. 2014;15(12):e538-48.

2. Björk J, Grubb A, Sterner G, Nyman U. Revised equations for estimating glomerular filtration rate based on the Lund-Malmö Study cohort. Scand J Clin Lab Invest. 2011;71(3):232-9.

3. Bossuyt X, Delforge M, Reynders M, Dillaerts D, Sprangers B, Fostier K, et al. Diagnostic thresholds for free light chains in multiple myeloma depend on the assay used. Leukemia. 2018;32(8):1815-8.

4. White-Al Habeeb NMA, Earle T, Spencer M, Blasutig IM. Evaluation of the N-latex serum free light chain assay on the Siemens BNII analyzer and agreement with The Binding Site FreeLite assay on the SPAPlus. Clin Biochem. 2018;51:90-6.

5. Yang Y, Han X, Zheng G, Cai Z. Comparison of two serum free light chain assays for the diagnosis of primary plasma cell malignant proliferative disease. Health Sci Rep. 2019;2(4):e113.

6. Lock RJ, Saleem R, Roberts EG, Wallage MJ, Pesce TJ, Rowbottom A, et al. A multicentre study comparing two methods for serum free light chain analysis. Ann Clin Biochem. 2013;50(Pt 3):255-61.

7. Yang Z, Harrison K, Park YA, Chaffin CH, Thigpen B, Easley PL, et al. Performance of the Sebia CAPILLARYS 2 for detection and immunotyping of serum monoclonal paraproteins. Am J Clin Pathol. 2007;128(2):293-9.

8. McCudden CR, Mathews SP, Hainsworth SA, Chapman JF, Hammett-Stabler CA, Willis MS, et al. Performance comparison of capillary and agarose gel electrophoresis for the identification and characterization of monoclonal immunoglobulins. Am J Clin Pathol. 2008;129(3):451-8.

9. Litwin CM, Anderson SK, Philipps G, Martins TB, Jaskowski TD, Hill HR. Comparison of capillary zone and immunosubtraction with agarose gel and immunofixation electrophoresis for detecting and identifying monoclonal gammopathies. Am J Clin Pathol. 1999;112(3):411-7.

10. Katzmann JA, Snyder MR, Rajkumar SV, Kyle RA, Therneau TM, Benson JT, et al. Long-Term Biological Variation of Serum Protein Electrophoresis M-Spike, Urine M-Spike, and Monoclonal Serum Free Light Chain Quantification: Implications for Monitoring Monoclonal Gammopathies. Clinical Chemistry. 2011;57(12):1687-92.

11. Salamatmanesh M, McCudden CR, McCurdy A, Booth RA. Monoclonal protein reference change value as determined by gel-based serum protein electrophoresis. Clinical Biochemistry. 2018;51:61-5.

12. Evliyaoglu O, van Helden J, Jaruschewski S, Imohl M, Weiskirchen R. Reference change values of M-protein, free light chain and immunoglobulins in monoclonal gammopathy. Clin Biochem. 2019;74:42-6.

13. Henriot B, Rouger E, Rousseau C, Escoffre M, Sebillot M, Bendavid C, et al. Prognostic value of involved/uninvolved free light chain ratio determined by Freelite and N Latex FLC assays for identification of high-risk smoldering myeloma patients. Clin Chem Lab Med. 2019;57(9):1397-405.

14. Kyle RA, Remstein ED, Therneau TM, Dispenzieri A, Kurtin PJ, Hodnefield JM, et al. Clinical course and prognosis of smoldering (asymptomatic) multiple myeloma. N Engl J Med. 2007;356(25):2582-90.

15. Dispenzieri A, Kyle RA, Katzmann JA, Therneau TM, Larson D, Benson J, et al. Immunoglobulin free light chain ratio is an independent risk factor for progression of smoldering (asymptomatic) multiple myeloma. Blood. 2008;111(2):785-9.

16. Waxman AJ, Mick R, Garfall AL, Cohen AD, Vogl DT, Stadtmauer EA, et al. Modeling the risk of progression in smoldering multiple myeloma. Journal of Clinical Oncology. 2014;32(15).

17. Sorrig R, Klausen TW, Salomo M, Vangsted AJ, Ostergaard B, Gregersen H, et al. Smoldering multiple myeloma risk factors for progression: a Danish population-based cohort study. Eur J Haematol. 2016;97(3):303-9.

18. Lakshman A, Rajkumar SV, Buadi FK, Binder M, Gertz MA, Lacy MQ, et al. Risk stratification of smoldering multiple myeloma incorporating revised IMWG diagnostic criteria. Blood Cancer J. 2018;8(6):59.

19. Hajek R, Sandecka V, Spicka I, Raab M, Goldschmidt H, Beck S, et al. Identification of patients with smouldering multiple myeloma at ultra-high risk of progression using serum parameters: the Czech Myeloma Group model. Br J Haematol. 2020;190(2):189-97.

20. Mateos MV, Kumar S, Dimopoulos MA, Gonzalez-Calle V, Kastritis E, Hajek R, et al. International Myeloma Working Group risk stratification model for smoldering multiple myeloma (SMM). Blood Cancer J. 2020;10(10):102.

21. Ravi P, Kumar S, Larsen JT, Gonsalves W, Buadi F, Lacy MQ, et al. Evolving changes in disease biomarkers and risk of early progression in smoldering multiple myeloma. Blood Cancer J. 2016;6(7):e454.

22. Fernandez de Larrea C, Isola I, Pereira A, Cibeira MT, Magnano L, Tovar N, et al. Evolving M-protein pattern in patients with smoldering multiple myeloma: impact on early progression. Leukemia. 2018;32(6):1427-34.

23. Wu V, Moshier E, Leng S, Barlogie B, Cho HJ, Jagannath S, et al. Risk stratification of smoldering multiple myeloma: predictive value of free light chains and group-based trajectory modeling. Blood Adv. 2018;2(12):1470-9.

24. Atrash S, Robinson M, Slaughter D, Aneralla A, Brown T, Robinson J, et al. Evolving changes in M-protein and hemoglobin as predictors for progression of smoldering multiple myeloma. Blood Cancer J. 2018;8(11):107.
